# Antifungal Resistance among Less Prevalent *Candida* Non-*albicans* and Other Yeasts versus Established and under Development Agents: A Literature Review

**DOI:** 10.3390/jof7010024

**Published:** 2021-01-04

**Authors:** Ana Espinel-Ingroff, Emilia Cantón, Javier Pemán

**Affiliations:** 1 Department of Medicine, VCU Medical Center, Richmond, VA 23298, USA; 2 Severe Infection Research Group, Health Research Institute Hospital La Fe, 46026 Valencia, Spain; canton_emi@gva.es (E.C.); peman_jav@gva.es (J.P.); 3 Microbiology Department, Hospital Universitari i Politècnic La Fe, 46026 Valencia, Spain

**Keywords:** non-prevalent *Candida*, antifungal resistance, new and established antifungal agents, *Candida*-non *albicans*, other yeast pathogens

## Abstract

Fungal diseases and antifungal resistance continue to increase, including those caused by rare or emerging species. However, the majority of the published in vitro susceptibility data are for the most common fungal species. We reviewed the literature in order to pool reference minimal inhibitory concentration (MIC) data (Clinical and Laboratory Standards Institute—CLSI and European Committee on Antimicrobial Susceptibility—EUCAST) for rare/non-prevalent *Candida* and other yeast species. MIC results were compared with those for *Candida albicans*, *C. glabrata*, and *C. krusei*. Data were listed for twenty rare and emerging *Candida* spp., including *C. auris*, as well as two *Cryptococcus* spp., two *Trichosporon* spp., *Saccharomyces cerevisiae* and five *Malassezia* spp. The best detectors of antimicrobial resistance are the breakpoints, which are not available for the less common *Candida* species. However, epidemiological cutoff values (ECVs/ECOFFs) have been calculated using merely in vitro data for both reference methods for various non-prevalent yeasts and recently the CLSI has established ECVs for other *Candida* species. The ECV could identify the non-wild type (NWT or mutants) isolates with known resistance mechanisms. Utilizing these ECVs, we were able to report additional percentages of NWT, especially for non-prevalent species, by analyzing the MIC distributions in the literature. In addition, since several antifungal drugs are under development, we are listing MIC data for some of these agents.

## 1. Introduction

Fungal infections are associated with high mortality and morbidity rates, especially among patients with invasive candidiasis and other systemic infections [1,2]. In addition, severe infections are caused worldwide by other yeast species (e.g., *Cryptococus* spp.) and a variety of *Candida* non-*albicans* species among immunocompromised patients, including those suffering non-infectious complications [3]. More recently, antifungal resistance has been increasing among prevalent and less common yeasts and molds; some of these species can be innately resistant to the available agents which leads to refractory infections [1]. A classic example is infections caused by the emerging pathogen *Candida auris*, which has been reported to be in vitro and clinically resistant to most agents [1,2]. Owing to those facts, yeast infections contribute to the increasing antifungal resistance and the overall mortality rate (~32 to 45%); the successful treatment response rate is also non-satisfactory (~67.4%) [1]. Invasive candidiasis, including candidemia, is mostly caused by the prevalent species: *Candida albicans*, *C. glabrata*, *C. parapsilosis*, *C. tropicalis*, and *C. krusei* [4]; but severe infections can be caused by the non-prevalent *Candida* spp. (e.g., *C. guilliermondii*, *C. inconspicua*, *C. lusitaniae*, *C. lipolytica*, *C. norvegensis*, *C. orthopsilosis*, *C. metapsilosis*, and *C. rugosa* among others (Table 1). Included in the non-*Candida* species, both *Cryptococcus neoformans* and *C. gattii* have been known to cause severe infections and outbreaks among immunocompromised patients [5]. About one million worldwide cases of HIV-associated cryptococcosis have been reported per year. Among other yeasts, the most common reports of in vitro data are for the five prevalent species and recently for *C. auris*. However, few reports of in vitro data are available for the less prevalent fungal species by either commercial or reference methods [6,7].

The best and more reliable detectors of microbial resistance are the breakpoints (BPs). The Clinical and Laboratory Standards Institute (CLSI) and the European Committee on Antimicrobial Susceptibility Testing (EUCAST) have developed reference methods and established BPs for the most common *Candida* spp., the triazoles and echinocandins [8,9,10]. Based solely on MIC (minimal inhibitory concentration) or MEC (minimal effective concentration) distributions, method-specific epidemiological cutoff values (ECVs/ECOFFs) were defined for a variety of yeast and mold species for susceptibility testing by both reference methods [9,10]. The ECV is another resistance detector and recently the CLSI has updated the M59 document by providing ECVs for some of the less prevalent *Candida* spp. [9].

The impact of azole and echinocandin resistance led to the genetic analyses of various triazole and echinocandin mutational mechanisms of resistance mentioned above. Data for individual mutant isolates are available in the literature for single strains of the most prevalent species. It is the role of the ECV to detect these mutants or non-wild type strains (NWT, defined as potentially harboring known resistance mechanisms) from the WT isolates (without resistance mechanisms) [9]. It is fortuitous that the CLSI has updated the M59 document including ECVs for some of the less prevalent *Candida* spp., *C. neoformans* and *C. gattii,* and that evaluations of various new antifungal agents under development have provided in vitro data for a variety of fungal species. Therefore, these advances have allowed a more comprehensive evaluation of the antifungal susceptibility of less prevalent species including *C. auris*. All these factors have facilitated the completeness of this review.

The main purpose of the review was to search the literature (Google Scholar, GetCitted, PubMed) for reports that included in vitro data for less prevalent, three prevalent *Candida* spp. (*C. albicans*, *C. glabrata* and *C. krusei*) and other yeasts versus both established and under development antifungal agents. This search provided sufficient data to: (1) compare the patterns of antifungal susceptibility among prevalent and non-prevalent *Candida* spp. using the available ECV for each species and established agent (Table 1); (2) to obtain percentages of WT and NWT for species/agent combinations (Table 2); (3) to summarize MICs for *C. auris* (Table 3); (4) to compile MIC data for individual triazole and echinocandin *Candida* mutants (Table 4); (5) to list published in vitro data for isolates of 10 non-*Candida* genera/species: two *Cryptococcus* spp., two *Trichosporon* spp., *Saccharomyces cerevisiae* and five *Malassezia* spp. (Table 5). Data for the following new agents were listed: the oral glucan synthase inhibitor manogepix (fosmanogepix (APX001; E1210), the two new echinocandins rezafungin (CD101, Cidara Therapeutics, San Diego, CA, USA) and ibrexafungerp (SCY-078, Scynexis, Inc., Jersey City, NJ, USA), and the Cyp51 inhibitor VT-1598. Since interest has been focused lately on the further development of the chitin synthase inhibitor nikkomycin, the search included that agent [2,3]. Although olorofim was included in the search (formerly F901318), no data were available due to its little or no activity against *Candida* spp. or other yeasts.

## 2. Review Guidelines

We searched the literature from ~2000 to the current time, especially the last five years, for MIC data for yeast species by the two reference methods (CLSI and EUCAST) and both established and/or the five new agents listed above. We also reviewed reports where MICs for individual NWT (mutants) and WT isolates of *Candida* spp. were listed and provided the specific echinocandin and/or triazole resistance mechanism. Data were not listed unless the isolates were identified to the species level, or where several species were pooled together as *Candida* spp., or for less than 10 isolates/species/agent, or data by any of the available commercial methods. Distributions for less than 10 isolates could be bimodal or truncated. Although some of the publications included caspofungin MICs, we did not include those results, since data for this agent and *Candida* spp. have been found to be unreliable (interlaboratory modal variability); also reference ECVs are not available for any *Candida* spp. [11]. The new taxonomic names for some of the species evaluated are given under the footnotes. When the distribution was reported and the ECV is available, we were able to calculate the percentage of isolates above the ECV (NWT isolates). Some reports provided ECVs for the new agents as two dilutions above the mode, but those ECVs were based on data from single laboratories and not listed. Since data are presented by both reference methods, the EUCAST data have been identified in the tables. 

## 3. Antifungal Susceptibility for Three Prevalent and Thirteen Rare *Candida* spp.

Table 1 depicts a total of 6,040 MICs for *C. albicans*, *C. glabrata* and *C. krusei* as well as for 14 non-prevalent *Candida* spp. versus six established antifungal agents (representing the different available classes) and MIC data for three of the agents under development: rezafungin (RZF), ibrexafungerp (IBX) and manogepix (MGX). We listed the reported range, the mode (underlined) or the most frequent MIC in the distribution) or the MIC90 for each species/agent. When the mode was not reported, but the distribution was, the mode was defined (most frequent MIC in the distribution) (Table 1 and Table 3). As discussed below, the % of NWT isolates are depicted in Table 2. These percentages (MICs above the ECV) were calculated by using the available MIC distribution for the species/agent or those reported by the authors.

## 4. Amphotericin B

Although there are no CLSI BPs for this agent and any fungal pathogen, ECVs have been established by both reference methods for certain species [9,10] (Table 1 and Table 2) [13,14,15,16,17,18,19,20,22,23,24,26]. In most instances, the highest MIC in the distribution is 1 µg/mL, with the exception of one distribution each of *C. guilliermondii* (0.03% NWT isolates) and *C. lusitaniae* (0% NWT) (Table 1 and Table 2) [14,22,23,25]. As per the ECV definition, all these isolates will be considered WT. Since the mechanisms of amphotericin B resistance have not been clearly elucidated, data for mutants are not available. *C. lusitaniae* and amphotericin B will be discussed in some detail with the information presented in Table 4.

## 5. Triazoles

As expected, more fluconazole and voriconazole than posaconazole data were reported for some of the less prevalent *Candida* species (Table 1). ECVs have not been defined for isavuconazole and any yeast species and very little data were found for this agent among the publications for the new agents; the exceptions being the data for isolates of *C. auris*, the two *Cryptococcus* species listed, *S. cerevisiae* and the set of data for isolates analyzed for genetic mechanisms of resistance (Table 3, Table 4 and Table 5). Mutants are present in any MIC distribution when the MIC range is above the ECV [9]; the percentages of NWT isolates are discussed below and summarized in Table 2. If not provided by the authors, these percentages are based on the reported MIC distributions and the ECVs for the particular agent/species combination.

## 6. Fluconazole

Although the number of fluconazole MICs for *C. albicans* was substantial in most of the reports and by both methods, the MIC profiles were generally good with low modes/MIC_90_s as well as percentages of fluconazole mutants (0 to 5.6%, CLSI/EUC data) (Table 1 and Table 2) [13,14,18,19,20,22,25,26]. MIC distribution dependent, the percentage of fluconazole mutants was higher (~11%) for *C. glabrata* [13,14,19]. Similar results were obtained for *C. guilliermondii* (2.4–8.4%), *C. lusitaniae* (9.2–12.7%), *C. metapsilosis* (3.3%) and *C. orthopsilosis* (4.4–10%), but overall lower for *C. kefyr* (1.9–5.6% [14,18,19,20,25] (Table 2). High fluconazole modes also were reported for *C. norvegensis* [25,26]. Some of these values are based on a small number of isolates. Among the non-prevalent *Candida* spp., for which ECVs have not been established, high fluconazole MICs were reported for *C. inconspicua* (modes:16 and >64 µg/mL) and *C. rugosa* (range: 0.12–16 µg/mL) [18,20,26].

## 7. Posaconazole and/or Voriconazole

In general, and as expected, posaconazole and voriconazole MICs were low for most *Candida* spp. (Table 1 and Table 2) [13,14,15,16,19,20,22,23,24,25,26]. Although BPs have not been defined for posaconazole, CLSI ECVs are available for all non-prevalent species listed in Table 2. Only the EUCAST ECV for *C. guilliermondii* and posaconazole has been defined among the non-prevalent *Candida* spp. [9,10]. The percentages of CLSI NWT for posaconazole were 2 and 23% for *C. guilliermondii* and 9.6 and 35.9% for *C. lusitaniae*; there were 1.5 and 10% NWT isolates for *C. orthopsilosis* and voriconazole (Table 2) [14,19,20,22,25]. These discrepancies could be due to the number of isolates evaluated and/or the use of different endpoints since some of the percentages were obtained by the authors.

## 8. Isavuconazole

Isavuconazole data were found mostly for *C. auris* and those will be discussed below as well as the reported data for well characterized triazole mutants and WT isolates and non-*Candida* spp. (Table 3 and Table 4). Most isavuconazole distributions are very irregular (truncated and/or bimodal), which is the reason that ECVs for this agent and *Candida* spp. are not yet available as far as we know.

## 9. Echinocandins

The CLSI and EUCAST echinocandin ECVs have been defined for a variety of prevalent species (Table 1 and Table 2) [9,10]. As mentioned above, the CLSI established echinocandin ECVs for five of the non-prevalent species listed in Table 1 and Table 2: *C. guilliermondii*, *C. lusitaniae*, *C. kefyr*, *C. metapsilosis*, and *C. orthopsilosis* [9]. The percentages of NWT isolates for *C. albicans* were: 0.2% (anidulafungin) and 0.7, 1.3 and 12.5% (micafungin). The percentages of NWT for *C. glabrata* were: 3.4 to 5% (anidulafungin) and 3.9 to 5% (micafungin) [12,13,14,18,19] (Table 2). With the exception of one EUCAST MIC distribution [13], the others have >100 isolates [12,14,18,19]. In contrast to the triazoles, MICs above the ECVs were not observed among the five non-prevalent species for which ECVs are available [12,14,15,17,18,19,24] (Table 2).

## 10. Agents under Development

MICs for the two new echinocandins, rezafungin and ibrexafungerp, were mostly ≤1 µg/mL for the three prevalent species [13,14,15,16,17,18,19]. The exceptions were rezafungin MICs for *C. glabrata* and manogepix MICs for *C. krusei* [14,15,17,21] (Table 1). The less prevalent species were evaluated by both methods as follows: rezafungin [17,18,19,21], ibrexafungerp [24] and manogepix [14,15]. In general, only a few MICs were >1 µg/mL among isolates of *C. guillermondii* (rezafungin and ibrexafungerp) [18,24], *C. lusitaniae* and *C. pelliculosa* (ibrexafungerp) [24], and *C. orthopsilosis* (rezafungin) [19] (Table 1). In some publications, ECVs were calculated as two concentrations above the mode. According to the CLSI ECV criteria, ECVs should be defined with data from at least three independent laboratories and for ≥100 isolates/species. The modal evaluation requires that modes from the different/species/agent should be within two drug concentrations. So, we did not enter these ECVs in any of the tables.

## 11. *Candida auris*

We have gathered reported MIC data for 938 isolates of *C. auris* versus both established and four new agents; data for VT-1598 also were found in the literature for this species (Table 3) [13,16,17,18,28,29,30,31,32,33,34]. The number of isolates tested in each report ranged from 13 to 350. The main problem with the distributions was that some of them were truncated and/or had more than one mode. As expected, fluconazole and voriconazole were evaluated in most studies and high modes were reported (≥32 µg/mL and mostly ≥1 µg/mL, respectively). The in vitro activity of the other triazoles was superior, including isavuconazole (modes ≤0.25 µg/mL) [13,28,29,30]. Overall, good activity was also observed against this species with both established and new echinocandins (modes ≤0.5 µg/mL) [13,17,18,28,29,30,31,32] and manogepix (≤0.12 µg/mL) [16,31,32,33]. Amphotericin B MICs had wide ranges, with MICs >1 µg/mL in 5/8 of the distributions listed in Table 3. We found one publication that reported VT-1598 CLSI MICs for 100 isolates of this species (0.03–>8 µg/mL and 0.25 µg/mL, MIC range and MIC90, respectively) [34]. Two publications also described data from murine models of disseminated *C. auris* infections, where animals were treated either with VT-1598 or rezafungin [34,35]. VT-1598 led to survival and reduction in fungal burden in both brain and kidney and similar results were observed when the animals were treated with caspofungin, but not with fluconazole [34]. In the other study, results were also satisfactory when the immunocompromised mice were treated with rezafungin [35]. It is interesting that although *C. auris* isolates were evaluated in different laboratories and most of them came from different geographical areas, the majority originated in India. The data collected for *C. auris* harboring genetic mechanisms of resistance are depicted in Table 4. For this species, the susceptibility testing is important due to the variety of MIC data with the established and new echinocandins.

## 12. Data for Isolates with Triazole or Echinocandin Resistant Genetic Mechanisms

In Table 4, we have summarized MICs for 458 genetically analyzed *C. albicans* and *C. glabrata* and 52 *C. auris* mutants and WT isolates; a set of controversial data for *C. lusitaniae* is also listed. For the triazoles, the NWT isolates were reported as having modifications of either the ABC or MFS transporters, or CDR gene overexpression, or individual deletions of the zinc cluster transcription factor genes *PDR1* and *UPC2A*, or ERG11 gene point mutations. The echinocandin *Candida* mutants harbored *fks* mutations [14,15,17,21,24,27,36,37,38]. Some reports were for resistant/susceptible isolates, respectively, as well as for 101 and *C. glabrata* mutants and 55 WT isolates, respectively, are listed in Table 4. Available CLSI or EUCAST ECVs were also listed [9,10]. Overall, the MIC ranges for the mutants were wider than those for the WT isolates, including those for the new agents. In addition, an MIC overlap was observed among mutant and WT isolates (darker shade in Table 4). An overlap is frequently seen, and it is clear that some mutations do not affect the phenotype to the same extent that others do for some of those isolates; it could be that some mutations might actually be simple (silent) polymorphisms or that the overlap is due to interlaboratory variability. It is important to remember that the ECV does not predict response to therapy, a non-WT may or may not respond to therapy [9].

Regarding the 52 *C. auris* mutants found in two reports [17,29] and shown in Table 4, the MICs are also much higher (wider ranges) than those in Table 3, including the data for both established echinocandins and rezafungin. However, the number of isolates was small in the latter report (eight isolates) [17].

The data for *C. lusitaniae* included amphotericin B MICs for five isolates evaluated in candidemia murine models responding to treatment with this agent (CLSI MIC range: 0.5–2 µg/mL from different laboratories) and for 38 isolates (CLSI MIC range: 0.03–0.5 µg/mL) from patients with no history of amphotericin B therapy [39]. The CLSI ECV is 2 µg/mL for this species and agent [9]; the EUCAST ECV is 0.5 µg/mL [10]. There is a potential overlap between WT and NWT isolates, or the CLSI ECV is too high to capture all the NWT strains. This can be observed in the amphotericin B data in Table 1 for *C. lusitaniae*, where the MIC range is 0.06–2 µg/mL for 1207 isolates. The Etest ECV is 0.5 µg/mL (data not shown in Table 4). If the Etest is a better detector of amphotericin B therapy, that would be helpful since spontaneous resistance with this agent can be developed during therapy. A similar statement can be found in the current M59 document [9].

## 13. Non-*Candida* spp.

### 13.1. Cryptococcus *spp.*

In Table 5, we have listed the MICs for a variety of yeast species, including *C. neofomans* var. *grubii* and *C. gattii* [14,19,25,27,40,41]. In one study, the antifungal susceptibility of the different serotypes of both species was provided (28, 25, and 33 isolates of serotypes A, B, and AD of *C. neoformans* and 30 and 12 isolates of *C. gattii*, respectively) [27,40]. However, we only displayed the data for the two pools of *C. neorformans* and *C. gatti* serotypes, since the MIC ranges of the different serotypes were similar [40]. Three publications included data of three new agents (rezafugin, manogepix and VT-1598) versus *C. neoformans* [14,19,27]. As expected, MICs for the latter species were high for the established echinocandins and rezafungin (2–>8 µg/mL) and low for manogepix (0.03–2 µg/mL). The geometric means of VT-1598 were lower (0.016 and 0.039) than those for fluconazole (1.89 and 2.71) and *C. neoformans* vs. *C. gattii* [27].

### 13.2. Trichosporon *spp.* and S. cerevisiae

Relatively low MICs were reported for both *Trichosporon* spp. and *S. cerevisiae* with the established agents; the exceptions are some elevated fluconazole MICs [42] (Table 5). Although no data were available for the new agents and *Trichosporon* spp., low echinocandin MIC data (including rezafungin) were reported for *S. cerevisiae* [17,18,25,41,42,43].

In general, *S. cerevisiae* infections are treated with the triazoles and the advent of isavuconazole was expected to be beneficial for these species.

### 13.3. Malassezia *spp.*

With the larger amount of immunosuppressed patients, the incidence and severity of dermatological and systemic infections caused by *Malassezia* spp. began to be reported [44,45,46]. However, due to the specific and complex nutritional requirements, neither EUCAST nor the CLSI has established standard guidelines for testing these species. A variety of media such as Christensen’s urea (measures metabolic activity) and supplemented RPMI 1640 broths have been evaluated [44]. We have summarized published MICs for the triazoles, including ketoconazole, and amphotericin B in Table 5. To our knowledge, these strains have not been evaluated with the new agents. Based on pharmacokinetic (PK) values, voriconazole MICs of 1 µg/mL and 4 µg/mL have been correlated with clinical response or non-response, respectively; the latter also applies to itraconazole MICs of 1 µg/mL. However, a standardized method would allow the classification of these isolates as WT and non-WT according to ECVs.

## 14. Nikkomycin

This agent has been evaluated for the treatment of infections caused by endemic fungi, especially *Coccidioides* spp. Some early publications described its in vitro activity in combination with fluconazole and/or itraconazole for *Candida* spp. and *C. neoformans* [47]. More recently, its activity has been evaluated in combination with the echinocandins against *fks C*. *albicans* mutants (data not listed in Table 1) [48].

## 15. Conclusions

Abundant MIC data for common and rare yeast species are now available for established and new agents obtained by both standard methods. Based on these advances, we compiled 6,040 published MICs for rare and three common *Candida* spp. (Table 1), 938 *C. auris* isolates (Table 3); 900 isolates of *C. neoformans* and *C. gattii* and 1,136 isolates of other yeast species (Table 5). Using the recently established CLSI ECVs for some non-prevalent *Candida* spp., we provided estimated percentages of NWT among 1,569 isolates of less prevalent species (Table 2). Our search also revealed a good amount of triazole and echinocandin MIC data for single and genetically defined WT and NWT isolates of *C. albicans* (225) and *C. glabrata* (156), as well as for 52 *C. auris* mutants (Table 4). Some of those summaries included MICs for rezafungin, ibrexafungerp, manogepix and VT-1598. Therefore, the data accumulated in the present literature review could be useful in the clinical laboratory in identifying potential resistance among the less prevalent yeast species.

## Figures and Tables

**Table 1:** **Reference minimal inhibitory concentrations (MICs) of new and established agents for 6,040 isolates of less prevalent and three common *Candida* spp. associ-ated with systemic diseases.**

Species (no. isolates)	Antifungal agent, MIC ranges, (Mode or MIC90)									
	AMB	FLU	VOR	POS	AND	MCF	RZF	IBX	MGX	Ref.
***C. albicans***										
**CLSI/EUCAST ECVs**	**2/1**	**0.5/0.5**	**0.03/0.03**	**0.06/0.06**	**0.12/0.03**	**0.03/0.01**	**NA**	**NA**	**NA**	[9,10]
(159) (16)	0.06–0.25 (NM)	0.12–0.25 (NM)	<0.01 (NM)		<0.01 (NM)	0.01–0.06 (0.01)*		0.03–0.12 (0.06)		[12,13] (EUCAST)
(414)	0.25–1 (1)	≤0.12–8 (0.25)	<0.01–0.12 (0.01)		<0.01–1 (0.03)	<0.01–2 (<0.01)			<0.01–0.03 (<0.01)	[14]
(402)	0.06–0.5 (0.25)		<0.01–>4 (<0.01)		<0.01–0.06 (<0.01)	<0.01–0.12 (0.01)			<0.01–0.25 (0.01)	[15] (EUCAST)
(218)	0.06–0.5 (0.25)	0.03–4 (0.25)	<0.01–0.06 (<0.01)		<0.01–0.01 (<0.01)	<0.01–0.03 (<0.01)			<0.01–0.03 (<0.01)	[16] (EUCAST)
(569)	0.06–0.5 (0.25)	0.03–>64 (0.12)			<0.01–0.25 (<0.01)	<0.01–2 (0.01)	0.01–1 (0.06)			[17] (EUCAST)
(125)	0.12–2 (0.5)	≤0.12–>32 (0.12)			<0.01–0.06 (<0.01)	<0.01–0.25 (0.01)	<0.01–0.12 (0.03)			[18]
(251)	0.5–1 (1)	≤0.12–>128 (0.25)	<0.01–>8 (0.01)	<0.01–>8(0.06)	<0.01–0.12 (0.06)	<0.01–0.03 (0.03)	<0.01–0.12 (0.06)			[19]
										
***C. fabianii*** (15)	0.25–1 (0.5)	0.12–2 (0.5)			0.01–0.25 (0.06)	0.06–0.5 (0.06)	0.03–0.12 (0.06)			[18]
										
***C. famata*** (49,53,45)		0.12–4 (0.25)	<0.01–0.25 (0.03)	0.01–0.5 (0.06)						[20]
***C. glabrata***										
**CLSI/EUCAST ECVs**	**2/1**	**8/16**	**0.25/1**	**1/1**	**0.25/0.06**	**0.03/0.03**	**NA**	**NA**	**NA**	[9,10]
(16)	0.03–0.5 (0.25)	2–4 (4)*	0.03–0.12 (0.06)		0.01–0.03 (0.03)*	0.01–0.03 (0.01)*		0.25–0.5 (0.25)*		[13] (EUCAST)
(179)	0.06–0.5 (0.5)	0.5–>32 (4)	0.01–>4 (0.06)		<0.01–0.25 (0.01)	<0.01–0.12 (0.01)			<0.01–0.25 (0.06)	[16] (EUCAST)
(25)					<0.01–4 (0.06)		0.01–2 (0.06)			[21]
(328)	0.03–1 (0.25)	0.5–>64 (4)			<0.01–1 (0.03)	<0.01–0.5 (0.01)	0.03–2 (0.12)			[17] (EUCAST)
***C. guilliermondii***										
**CLSI/EUCAST ECVs**	**2/0.5**	**16/16**	**NA/NA**	**0.5/0.25**	**8/NA**	**2/NA**	**NA**	**NA**	**NA**	[9,10]
(24)	>2 (0/24)	>8 (4/24)	>0.25 (4/17)	>0.5 (4/17)						[22]
(47) (373, 369, 298)	0.25–2 (0.5)	0.12–>64 (2)	<0.01–>8 (0.03)	<0.01–2 (0.12)						[20,23]
(9)	0.12–1 (1)	2–>64 (>64)	0.03–4 (4)		0.25–1 (1)	0.12–0.5 (0.5)			<0.01–0.06 (0.06)	[15] (EUCAST)
27	0.25–1 (0.5)	1–32 (2)			0.25–2 (1)	0.5–2 (1)	0.5–2 (1)			[18]
(23)					1–4 (2)	0.5–2 (1)		1–4 (2)		[24]
(376, 357)	0.06–16 (0.25)	<0.12–>64 (4)								[25]
										
***C. inconspicua*** (41)	0.06–1 (0.5)	8–>32 (16)			<0.01 (0.01)	<0.01–0.12 (0.03)	0.01–0.06 (0.06)*			[18]
(168)	(1)	(>64)	(1)	(0.5)	(0.12)	(0.12)				[26] (EUCAST)
										
***C. kefyr***										
**CLSI/EUCAST ECVs**	**2/1**	**1/1.0**	**NA**	**0.5/NA**	**0.25/NA**	**0.12/NA**	**NA**	**NA**	**NA**	[9,10]
(13, 12, 12)	NA	>1 (1/13)	>0.01 (2/12)	>0.25 (1/12)	NA	NA				[22]
(36, 34, 29, 13, 13)	0.5–1 (1)	<0.12–0.5 (0.25)	<0.016–0.12 (0.01)	0.03–0.5 (0.12)	0.03–0.12 (0.12)				0.06–0.5 (0.06)*	[14,20]
(12)	0.25–1 (0.5)	0.25–8 (0.5)	<0.01–0.06 (0.01)		0.01–0.06 (0.03)	0.01–0.12 (0.06)			0.12–>0.5* (0.5)	[15] (EUCAST)
(136)						0.01–0.12 (0.03)				[12] (EUCAST)
(52)	0.25–1 (0.5)	0.12–4 (0.12)			<0.01–0.12 (0.03)	<0.01–0.12 (0.06)	0.01–0.25 (0.06)			[18]
(12)					0.03–0.06 (0.06)	0.03–0.12 (0.06)		0.06–1 (0.5)*		[24]
***C. krusei***										
**CLSI/EUCAST ECVs**	**2/1**	**NA/128**	**0.5/1**	**0.5/0.5**	**0.25/0.06**	**0.25/0.25**	**NA**	**NA**	**NA**	[9,10]
(127)						<0.01–0.25 (0.06)*				[12] (EUCAST)
(43)	1–2 (1)	16–64 (32)	0.12–1 (0.5)	0.06–0.5 (0.5)	0.01–0.12 (0.12)	<0.01–0.12 (0.12)			>2 (>2)	[14]
(54)	0.5–1 (1)	8–>64 (NA)	0.12–>4 (0.5)		0.01–0.25 (0.06)	0.06–4 (0.25)			0.25–>0.5 (>0.5)	[15] (EUCAST)
(20)					0.01–2 (0.06)		0.01–1 (0.06)			[21]
***C. lipolytica*** (10)	0.12–0.5(0.25)	0.5–2(0.5)			0.03–0.12(0.12)*	0.06–1(1)*	0.03-0.06(0.06*)			[18,25]
(42), (32), (38)	0.03–2(0.5)*	0.5–>64(4)	0.03–4(0.06)							
***C. lusitaniae***										
**CLSI/EUCAST ECVs**	**2/0.5**	**1/NA**	**NA**	**0.06/NA**	**1/NA**	**0.5/NA**	**NA**	**NA**	**NA**	[9,10]
(14, 19)	>2 (0/19)	>2 (3/19)	>0.03 (1/14)	>0.12 (2/14)						[22]
(71) (574, 521, 142)	0.06–2 (1)	<0.12–64 (0.5)	<0.03–0.25 (0.01)	<0.01–1 (0.01)						[20,23]
(23)						0.3–0.12 (0.06)				[12] (EUCAST)
(12)	0.06–1 (0.25)	0.25–0.5 (0.5)	<0.01 (0.01)		0.03–0.12 (0.06)	0.03–0.25 (0.12)			<0.01 (0.01)	[15] (EUCAST)
(46)	0.12–1 (0.5)	0.06–32 (0.25)			0.01–0.25 (0.03)	0.01–0.5 (0.12)	0.01–0.5 (0.12)			[18]
(20)	0.06–1 (0.12)	0.25–32 (0.5)				0.03–0.5 (0.06)	0.12–0.5 (0.12)*			[17] (EUCAST)
(22)					0.01–1 (0.5)	0.01–0.5 (0.25)		0.5–4 (2)		[24]
(39)	0.25–1 (1)	<0.12–64 (2)	<0.01–0.5 (0.01)	0.03–0.25 (0.12)	0.12–0.5 (0.5)	0.06–0.5 (0.25)			0.01–0.5 (0.12)*	[14]
(452, 529, 327, 81)	0.03–2 (0.25)	<0.12–>64 (0.5)	<0.03–4(<0.03)*		<0.01–0.5 (0.06)					[25]
***C. metapsilosis***										
**CLSI ECVs**	**1**	**4**				**1**	**NA**	**NA**	**NA**	[9]
(15)	0.2–1 (0.5)*	0.5–16 (1)*			0.12–0.5 (0.25)	0.06–0.5 (0.25)	0.25–0.5 (0.5)			[18]
(31, 30, 24, 14)	0.06–1 (0.25)	0.12–16 (1)	0.03–0.12 (0.03)		0.06–0.5 (0.25)					[25]
***C. norvegensis*** (15)	(2)	(>64)	(4)	(0.5)	(0.12)	(0.12)				[26] (EUCAST)
(20, 20, 13)	0.12–0.25 (0.25)*	8–64 (16)	0.03–0.25 (0.25)*							[25]
										
***C. orthopsilosis***										
**CLSI ECVs**	**2**	**2**	**0.12**	**0.25**	**2**	**1**	**NA**	**NA**	**NA**	[9]
(68, 66, 55)		0.12–8 (0.5)	<0.01–0.25 (0.01)	<0.01–0.12 (0.03)						[20]
(10)					0.25–2 (1)*	0.12–1 (1)*	0.12–2 (1)*			[19]
(15)	0.12–0.5 (0.25)	0.12–0.5 (0.25)			0.12–1 (1)	0.25–1 (0.5)	0.12–1 (1)			[18]
(15)					0.25–1 (1)	0.12–1 (0.5)		0.06–1 (0.5)		[24]
										
***C. pelliculosa*** (15)					<0.01–0.06 (0.01)	0.01–1 (0.06)*		0.25–2 (0.25)		[24]
										
***C. pulcherrima*** (10)	0.12–1 (0.5)	0.12–0.5 (0.25)			0.01–0.06 (0.01)	≤0.01–0.5 (0.06)	0.01–0.06 (0.03)			[18]
***C. rugosa*** (76, 59, 45)		0.12–16 (1)	<0.01–0.25 (0.03)	0.01–0.5 (0.06)						[20]
										
***C. sojae*** (10)	0.12–1 (0.5)	0.12–0.25 (0.25)*			<0.01–0.03 (0.03)	0.01–0.12 (0.06)	0.03–0.06 (0.06)*			[18]

AMB, amphotericin B, FLU, fluconazole, VOR, voriconazole, POS, posaconazole, AND, anidulafungin, MCF, micafungin, RZF, rezafungin, IBX, ibrexafungerp, and MGX, manogepix. Newly accepted taxonomic names or reclassifications are as follows: *C. famata* (*Debaryiomyces. hansenii*), *C. guilliermondii* (*Meyerozyma guilliermondii*)), *Candida inconspicua* (*Pichia cactophila*)*, C. kefyr* (*Kluyveromyces marxianus*), *C. krusei* (*Pichia kudriavzevii*), *C. lipolytica* (*Yarrowia lipolytica*), *Candida lusitaniae* (*Clavispora lusitaniae*), *Candida pelliculosa* (*Wickerhamomyces anomalus*), *C. norvegensis* (*Pichia norvegensis*). MICs were determined by Clinical and Laboratory Standards Institute (CLSI) or European Committee on Antimicrobial Susceptibility (EUCAST) broth microdilution methods [6,7]. NA: Not available. ECVs/ECOffs: epidemiological cutoff values [9,10]. Mode: most frequent MIC in the distribution; NM: no mode reported. MIC90: value at which 90% of the isolates were inhibited. * MIC distribution having more than one mode. Reference [27], only geometric means were reported for VT-1598.

**Table 2:** **Percentages of non-wild type (NWT) isolates among 2505 isolates of less prevalent and three common *Candida* spp. and established agents.**

Species (no. Isolates)	Antifungal agent, MIC ranges, (% NWT within each range)						
	AMB	FLU	VOR	POS	AND	MCF	Ref.
***C. albicans***							
**CLSI/EUCAST ECVs**	**2/1**	**0.5/0.5**	**0.03/0.03**	**0.06/0.06**	**0.12/0.03**	**0.03/0.01**	[9,10]
(159)						0.01–0.06 (1.3)	[12] (EUCAST)
(16)	0.06–0.25 (0)	0.12–0.25 (0)	<0.01 (0)		<0.01 (0)	<01–0.03 (12.5)	[13] (EUCAST)
(414)	0.25–1 (0)	0.12–8 (1.2)	<0.01–0.12 (0)		<0.01–1 (0.2)	<0.0–2 (0.7)	[14]
(125)	0.12–2 (0)	0.12–>32 (5.6)			<0.01–0.06 (0)	<0.01–0.25 (ND)	[18]
(251)	0.5–1 (0)	0.12–>64 (3.2)	<0.01–>8 (1.6)	<0.01–>8 (7.6)	<0.01–0.12 (0)	<0.01–0.03 (0)	[19]
***C. glabrata***							
**CLSI/EUCAST ECVs**	**2/1**	**8/16**	**0.25/1**	**1/1**	**0.25/0.06**	**0.03/0.03**	[9,10]
(152)						0.01–1 (3.9)	[12] (EUCAST)
(15)	0.03–0.5 (0)	2–4 (0)	0.03–0.12 (0)	NA	0.01–0.03 (0)	0.01–0.03 (0)	[13] (EUCAST)
(321)	0.25–2 (0)	<0.12–>64 (11.5)	<001–>4 (11.2)	0.03–>4 (7.2)	0.01–4 (3.4)	<0.01–2 (5)	[14]
(100)	0.5–2 (0)	0.25–>64 (11)	0.01–4 (11)	0.03–4 (1)	<0.01–4 (5)	<0.01–4 (5)	[19]
***C. guilliermondii***							
**CLSI/EUCAST ECVs**	**2/0.5**	**16/16**	**NA/NA**	**0.5/0.25**	**8/NA**	**2/NA**	[9,10]
(24)	No range	No range	No range	>0.5 (23)			[22]
(27)	0.25–1 (0)	1–32 (7.4)			0.25–2 (0)	0.5–2 (0)	[18]
(373,369,298)		0.12–>64 (2.4)	<0.01–>8 (NA)	<0.01–2 (2)			[20]
(47)	0.25–2 (0)						[23]
(376, 357)	0.06–16 (0.3)	<0.12–>64 (8.4)			0.06–4 (0)		[25]
***C. kefyr***							
**CLSI/EUCAST ECVs**	**2/1**	**1/1.0**		**0.5/NA**	**0.25/NA**	**0.12/NA**	[9,10]
(136)						0.01–0.12 (ND)	[12] (EUCAST)
(13)	0.5–1 (0)	0.12–0.5 (0)		0.06–0.25 (0)	0.03–0.12 (0)	0.03–0.06 (0)	[14]
(52)	0.25–1 (0)	0.12–4 (1.9)			<0.01–0.12 (0)	<0.01–0.12 (0)	[18]
(36,34,29)		0.12–8 (5.6)	<0.01–0.12	0.03–0.5 (0)			[20]
***C. krusei***							
**CLSI/EUCAST ECVs**	**2/1**	**NA/128**	**0.5/1**	**0.5/0.5**	**0.25/0.06**	**0.25/0.25**	[9,10]
(127)						0.01–0.25 (0)	[12] (EUCAST)
(43)	1–2 (0)	16–64 (ND)	0.12–1 (2.3)	0.06–0.5 (0)	0.01–0.12 (0)	<0.01–0.12 (0)	[14]
(53)	0.5–2 (0)	8–>32 (ND)			0.01–0.25 (0)	0.03–0.25 (0)	[18]
***C. lusitaniae***							
**CLSI/EUCAST ECVs**	**2/0.5**	**1/NA**	**NA**	**0.06/NA**	**1/NA**	**0.5/NA**	[9,10]
(23)						ND	[12] (EUCAST)
(39)	0.25–1 (0)	<0.12–64 (10.3)		0.03–0.5 (35.9)	0.12–0.25 (0)	0.06–0.5 (0)	[14]
(46)	0.12–1 (0)	0.06–32 (10.9)			<0.01–0.25 (0)	0.01–0.5 (0)	[18]
(574,142,521)		0.06–64 (9.2)	<0.02–0.25	<0.01–1 (9.6)			[20]
(71)	0.06–2 (0)						[23]
(452, 529)	0.03–2 (0)	<0.12–>64 (12.7)					[25]
***C. metapsilosis***							
**CLSI ECVs**	**1**	**4**	**0.06**	**0.25**	**0.5**	**1**	[9]
(15)	0.25–1 (0)	0.5–16 (ND)			0.12–0.5 (0)	0.06–0.5 (0)	[18]
(31, 30, 24, 14)	0.06–1 (0)	0.12–16 (3.3)	0.03–0.12 (4.2)		0.06–0.25 (0)		[25]
***C. orthopsilosis***							
**CLSI ECVs**	**2**	**2**	**0.12**	**0.25**	**2**	**1**	[9]
(15)	0.12–0.5 (0)	0.12–0.5 (0)			0.12–1 (0)	0.25–1 (0)	[18]
(10)	0.25–1 (0)	0.5–32 (10)	0.01–0.5 (10)	0.06–0.25 (0)	0.25–2 (0)	0.12–1 (0)	[19]
(68,66,55)		0.12–8 (4.4)	<0.01–0.25 (1.5)	<0.01–0.12 (0)			[20]

AMB, amphotericin B, FLU, fluconazole, VOR, voriconazole, POS, posaconazole, AND, anidulafungin, and MCF, micafungin. Newly accepted taxonomic names or reclassifications are as follows: *C. guilliermondii* (*Meyerozyma guilliermondii*), *C. kefyr* (*Kluyveromyces marxianus*), *C. krusei* (*Pichia kudriavzevii*), and *C. lusitaniae* (*Clavispora lusitaniae*). MICs were determined by CLSI or EUCAST (EUC) microdilution methods [6,7]. ECVs/ECOFFs, epidemiological cutoff values for detection of mutant (NWT) isolates by specific method, species, and agent [9,10]. NWT, non-WT or mutants. NA: not available.

**Table 3:** **MIC data by reference methods for 938 isolates of *Candida auris* and new and established agents.**

No. isolates	Antifungal Agent, MIC Ranges (Mode or MIC_90_)												Ref
	AMB	FLU	VOR	ITZ	ISA	POS	AND	MCF	RZF	IBX	MGX	VT-1598	
123	0.12–8 (0.5)	4–>64 (>64)*	0.03–16 (2)*	0.03–2 (0.12)*	0.01–4 (0.25)*	0.01–8 (0.01)*	0.01–8 (0.12)	0.01–8 (0.12)					[28]
350	0.12–8 (1)	1–>64 (>64)	0.03–16 (0.12)	0.03–16 (0.12)	<0.01–4 (0.03)*	<0.1–8 (<0.01)*	0.016–8 (0.5)	0.016–16 (0.12)					[29]
73	0.06–2 (0.25)	>64 (>64)	0.5–>8 (4)	0.06-0.5(0.25)	0.03–2 (0.12)	0.03–0.12 (0.12)	0.01–0.5 (0.06)	0.03–0.12 (0.06)					[30] (EUCAST)
122	0.5–1 (1)	0.5–>64 (>64)					0.01–>32 (0.06)	0.03–>32 (0.12)	0.06–16 (0.25)				[17] (EUCAST)
19	0.12–1 (1)	0.5–32 (>32)					0.03–0.5 (0.03)	0.06–2 (0.25)	0.03–0.25 (0.12)*				[18]
122,122	0.5–1 (1)	0.5–>64 (>64)	<0.01–4 (BM)*	NA	<0.01–2 (BM)*	NA	0.01–>32 (0.06)	0.03–>32 (0.12)		0.06–2 (0.5)	<0.01–0.12 (<0.01)		[13,16] (EUCAST)
27	0.5–4	8–>64					0.25–>16	0.06–>8			<0.01		[31]
16	0.5–8 (4)	1–64 (>64)	<0.01–1 (1)	<0.01–1 (1)		0.25–1 (1)	0.12–0.25 (0.25)	0.25–2 (2)			<0.01–0.06 (0.03)		[32]
13		2–>64 (>64)									<0.01–0.03 (0.12)		[33]
100												0.03–>8 (0.25)	[34]

AMB, amphotericin B; FLU, fluconazole; VOR, voriconazole; ITZ, itraconazole; ISA, isavuconazole; POS, posaconazole; AND, anidulafungin; MCF, micafungin; RZF, rezafungin; IBX, ibrexafungerp; MGX, manogepix; VT-1598. MICs were determined by CLSI or EUCAST broth microdilution methods [6,7]. NA: not available. Mode: most frequent MIC in the distribution; MIC90: value at which 90% of the isolates were inhibited. *MIC distribution having more than one mode. Reference [29], multi-drug resistant isolates; references [32] and [35] provided in vivo data.

**Table 4:** **MIC data by reference methods of new and established agents for *Candida* spp. isolates with known resistance mechanisms (NWT).**

Species (No. tested)	Antifungal Agent, MIC Range, (Mode or MIC_90_)											Ref.
	FLU	ITZ	POS	VOR	ISA	AND	MCF	RZF	IBX	MGX*	VT-1598**	
***C. albicans***												
**CLSI/EUCAST ECVs**	**0.5/0.5**	**NA/0.06**	**0.06/0.06**	**0.03/0.03**	**NA**	**0.12/0.03**	**0.03/0.01**					[9,10]
**NWT**												
**>ECV/No. tested**	22/25	NA	12/25	20/25	<0.04–8/25							[36]
(3, 21)	49.1**					0.01–1	0.06–2	0.25–1		<0.01	0.124**	[14,27]
(7)										<0.01–0.25		[15] (EUCAST)
(10)						0.12–2		0.12–1				[21]
(10)							0.03–4	0.12–2				[37]
(8)’						<0.01–1 (0.12)	0.01–2 (0.06)		0.06–2 (NM)			[24]
(11)	0.12–>64					0.01–0.25	0.06–2	0.25–1				[17] (EUCAST)
**Total 95**												
**WT**												
**<ECV/No. tested**	10/11	11/11	11/11	11/11	11/11							[36]
(48)	0.277**											[27]
(10)							<0.03 (<0.03)	<0.03 (<0.03)				[37]
(61)						<0.01–0.06 (0.01)	<0.01–0.06 (0.01)		0.03–0.25 (0.12)			[24]
**Total 130**												
***C. glabrata***												
**CLSI/EUCAST ECVs**	**8/16**	**4/2**	**1/1**	**0.25/1**	**NA**	**0.25/0.5**	**0.03/0.03**					[9,10]
**NWT**												
**>ECV/No. tested**	6/9	1/9	6/9	6/9								[36]
(10,8)	76.1**					0.12–4	0.06–1			<0.01–0.12	1.19**	[14,27]
(6)										0.06–0.12		[15] (EUCAST)]
(9)						0.06–4		0.06–2				[21]
(11)							0.06–4	0.12–4				[37]
(10)	1.0–>64					0.06–1	0.06–0.5	0.5–2				[17] (EUCAST)
(10)	32–>64	1–4	1–2	1–4							0.5–2	[38]
(28)						0.01–4(1)	<0.01–4 (0.06)		0.12–16 (1)			[24]
**Total 101**												
**WT**												
**<** **ECV/No. tested**	7/7	7/7	7/7	7/7	0.25–1							[36]
(9)							<0.03 (<0.03)					[37]
(39)						0.01–0.25 (0.06)	<0.01–0.03 (0.01)		0.25–1 (0.5)			[24]
**Total 55**												
***C. auris***												
												
**NWT**												
(44)	1–64 (64)	0.03–0.5 (0.5)	0.01–8 (2)	0.03–16 (16)	0.01–4 (2)							[29]
(8)	>256					4.0–>32	>32	8–16				[17] (EUCAST)
**Total 52**												
***C. lusitaniae***	**AMB**											
**CLSI/EUCAST ECVs**	2/0.5											[39]
**NWT MIC range**	0.5–2											
>ECV/No. tested	(0/5)											
**WT MIC range**	0.3–0.5											
<ECV/No. tested	38/38											

FLU, fluconazole; ITZ, itraconazole; POS, posaconazole; VOR, voriconazole; ISA, isavuconazole; AND, anidulafungin; MCF, micafungin; RZF, rezafungin; IBX ibrexafungerp; MGX, manogepix; VT-1598. MICs were determined by CLSI and EUCAST microdilution methods [6,7]. Newly accepted taxonomic name or reclassification is as follows: *Candida lusitaniae* (*Clavispora lusitaniae*). ECVs/ECOFFS: epidemiological cut off values. NWT (non-WT or mutants): resistance mechanisms for the triazoles and *Candida*, isolates having ABC, MFS transporters, CDR gene overexpression, or ERG11 gene point mutations and for the echinocandins, isolates having *fks* mutations. Values below the ECV, darker shade; ECVs have not been established for the new agents. Mode: most frequent MIC in the distribution; MIC90: value at which 90% of the isolates were inhibited. *MIC distribution having more than one mode. ** Geometric mean. References [21] and [24] have data for other mutants (<5 isolates including 1 isolate of *C. pelliculosa*). NA, not available.

**Table 5:** **MIC data by reference methods for 709 isolates of *Cryptococcus* spp. and other yeast species and new and established agents.**

Species (No. isolates)	Antifungal agent, MIC ranges, (mode or MIC_90_)									Ref.
	AMB	FLU	POS	ISA	AND	MCF	RZF	MGX	VT-1598	
***C. neoformans var. grubii***										
**CLSI/EUCAST ECVs**	**05/1**	**8/NA**	**0.25/0.5**							[9,10]
(30)	0.25–1(1)	0.5-4(4)	0.03–0.25(0.25)		2–>4 (>4)	4–>4 (>4)		0.03–2 (0.5)		[14]
(19)	0.5-1(1)	1-4(4)	0.03-0.25(0.25)		8–>8(>8)	8->8(>8)	8->8(>8)			[19]
(523, 517, 21)	0.06–4 (0.25)	<0.12–64 (4)			8–32 (16)					[25]
(36)		1.89							0.016	[27]
(86)	0.03–1 (0.5)	0.25–64 (4)	<0.01–0.5 (0.06)	<0.01–0.5 (0.06)						[40]
(158)		<0.12–16 (8)		<0.01–0.5 (0.25)						[41] (EUCAST)
***C. gattii***										
**CLSI/EUCAST ECVs**	**0.05/0.5**	**16/NA**	**1/NA**							[9,10]
(16)		2.71							0.039	[27]
(42)	0.25–1 (0.25)	0.5–32 (8)	<0.1–0.25 (0.12)	<0.01–0.25 (0.06)						[40]
										
***Trichosporon asahii***										
(34, 33,18)	0.25–8 (1)	0.25–16 (1)			4–32 (16)					[25]
(40)	0.125–8 (2)	0.25–>64 (2)	(0.06-0.5(0.25)	0.03-0.25(0.12)						[42]
										
***T. mucoides*** (10)	0.25–2 (2)	0.12–1 (1)	0.03–0.25 (0.25)	0.03–0.25 (0.25)						[42]
***S. cerevisiae***										
(15)	0.03–1 (0.25)	0.25–32 (8)			0.01–0.12 (0.06)	0.6–0.25 (0.12)	0.12–0.5			[17] (EUCAST)
(21)	0.25-1(0.5)	2-8(4)			<0.01–0.5(0.12)	0.12-0.5(0.25)	0.03–0.5(0.5)			[18]
(448, 612, 97)	0.03–2 (0.5)	<0.12–>64 (4)			0.01–2 (0.25)					[25]
(18)	0.25–1 (0.5)	0.12–16 (4)	0.125–1 (0.5)	0.03–1 (0.25)						[42]
(15)	0.03-0.06(0.03)	0.03-0.12(0.12)	0.03-0.06(0.03)		0.03–0.5(0.06)	<0.01–0.5(0.03)				[43]
										
	**FLU**	**VOR**	**ITZ**	**POS**	**KET**					
***Malassezia furfur***										
(39)	>64 (>64)	0.03–4 (1)	<0.03–4 (0.25)	0.01–2 (0.25)						[44]
(52)		0.03–1 (0.5)	0.03–0.5 (0.25)		0.03–1 (0.5)					[45]
(78)	>64(>64)	0.06-8(2)	0.03-8(1)	0.01-8(0.5)						[46]
***M. globosa*** (74)		0.03–>8 (>8)	0.01–>8 (>8)		0.03–1 (0.5)					[45]
***M. pachydermatis*** (62)	4–>64 (8)	0.01–0.5 (0.06)	<0.01–0.12 (<0.01)	<0.01–0.03 (<0.01)	0.01–>8 (1)					[46]
***M. restricta*** (16)		0.06–8 (2)	0.01–>8 (2)		0.01–8 (1)					[45]
***M. sympodialis*** (50)		0.01–1 (0.25)	0.01–2 (0.06)		0.01–4 (0.5)					[45]

AMB, amphotericin B; FLU, fluconazole; POS, posaconazole; ISA, isavuconazole; AND, anidulafungin; MCF, micafungin; RZF, rezafungin; IBX, ibrexafungerp; MGX, manogepix; VOR, voriconazole; ITZ, itraconazole; KET, ketoconazole. MICs were determined by CLSI or EUCAST (EUC) broth microdilution methods [6,7]; NA: not available. ECVs/ECOFFs: epidemiological cutoff values. Mode: most frequent MIC in the distribution; MIC90: value at which 90% of the isolates were inhibited. * MIC distribution having more than one mode. The VT-1598 data were reported as geometric mean values (50% inhibition) [25]. Reference [39] reported MIC data for four isolates of *T. inkin* and 7 isolates of *G. capitatum*. Ref. 45 is in the text, to describe testing conditions.

## Data Availability

No new data were created or analyzed in this study. Data sharing is not applicable to this article.
